# Supplementary material for: Systemic Characterization of the Gut Microbiota Profile after Single Mild Ischemic Stroke and Recurrent Stroke in Mice
Source: Biomedicines. 2024 Jan 16;12(1):195. doi: 10.3390/biomedicines12010195 (PMC10813150; doi:10.3390/biomedicines12010195)
Supplement: Supplementary file 1 [file biomedicines-12-00195-s001.zip › biomedicines-2774105-supplementary.pdf]

Supplimental Figure S1

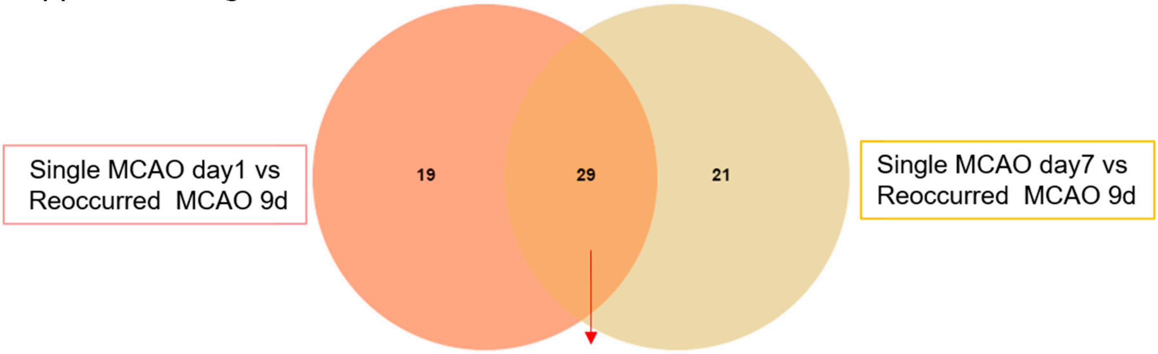


| Common Metabolic Pathway (continued from Figure 5B) | | | |
|---|---|---|---|
| 15 | **Superpathway of C1 Compounds Oxidation to CO2** (PWY-1882) | 22 | **Octane Oxidation** (P221-PWY) |
| | | 23 | **Aromatic Biogenic Amine Degradation** (PWY-7431) |
| 16 | **Superpathway of Bacteriochlorophyll a Biosynthesis** (PWY-5529) | 24 | **NAD Biosynthesis II** (from tryptophan) (NADSYN-PWY) |
| 17 | **Nylon-6 Oligomer Degradation** (P621-PWY) | 25 | **L-tryptophan Degradation to 2-amino-3-Carboxymuconate Semialdehyde** (PWY-5651) |
| 18 | **Ketogluconate Metabolism** (KETOGLUCONMET-PWY) | 26 | **Mycothiol Biosynthesis** (PWY1G-0) |
| 19 | **Meta Cleavage Pathway of Aromatic Compounds** (PWY-5430) | 27 | **Methyl Ketone Biosynthesis** (PWY-7007) |
| 20 | **Cob(II)yrinate a,c-diamide Biosynthesis II (late cobalt incorporation)** (PWY-7376) | 28 | **Mono-trans, Poly-cis Decaprenyl Phosphate Biosynthesis** (PWY-6383) |
| 21 | **Methanol Oxidation to Carbon Dioxide** (PWY-7616) | 29 | **Mycolyl-Arabinogalactan-Peptidoglycan Complex Biosynthesis** (PWY-6397) |

**Supplemental Figure S1.** The common altered metabolic pathway after single MCAO day 1 and day7 vs reoccurred MCAO day 9.
